# Analysis of Microalgal Density Estimation by Using LASSO and Image Texture Features

**DOI:** 10.3390/s23052543

**Published:** 2023-02-24

**Authors:** Linh Nguyen, Dung K. Nguyen, Thang Nguyen, Binh Nguyen, Truong X. Nghiem

**Affiliations:** 1 Institute of Innovation, Science and Sustainability, Federation University Australia, Churchill, VIC 3842, Australia; 2 College of Agriculture and Life Science, Chonnam National University, Gwangju 61186, Republic of Korea; 3 Department of Engineering, Texas A&M University–Corpus Christi, Corpus Christi, TX 78412, USA; 4 Department of Automation and Control Engineering, Thuyloi University, Hanoi 116705, Vietnam; 5 School of Informatics, Computing, and Cyber Systems, Northern Arizona University, Flagstaff, AZ 86011, USA

**Keywords:** microalgae, microalgal density, LASSO, image texture features, image processing, algal monitoring

## Abstract

Monitoring and estimating the density of microalgae in a closed cultivation system is a critical task in culturing algae since it allows growers to optimally control both nutrients and cultivating conditions. Among the estimation techniques proposed so far, image-based methods, which are less invasive, nondestructive, and more biosecure, are practically preferred. Nevertheless, the premise behind most of those approaches is simply averaging the pixel values of images as inputs of a regression model to predict density values, which may not provide rich information of the microalgae presenting in the images. In this work, we propose to exploit more advanced texture features extracted from captured images, including confidence intervals of means of pixel values, powers of spatial frequencies presenting in images, and entropies accounting for pixel distribution. These diverse features can provide more information of microalgae, which can lead to more accurate estimation results. More importantly, we propose to use the texture features as inputs of a data-driven model based on L1 regularization, called least absolute shrinkage and selection operator (LASSO), where their coefficients are optimized in a manner that prioritizes more informative features. The LASSO model was then employed to efficiently estimate the density of microalgae presenting in a new image. The proposed approach was validated in real-world experiments monitoring the *Chlorella vulgaris* microalgae strain, where the obtained results demonstrate its outperformance compared with other methods. More specifically, the average error in the estimation obtained by the proposed approach is 1.54, whereas those obtained by the Gaussian process and gray-scale-based methods are 2.16 and 3.68, respectively

## 1. Introduction

There has been increasing interest in culturing microalgae at a large scale for producing food, pharmaceutical products, and biodiesel [1]. More specifically, it was shown that some microalgae can provide polysaccharides, vitamins, β-carotene, long-chain polyunsaturated fatty acids, and antioxidants [2,3]. In fact, some microalgal species are used as aquafeed for molluscs, crustaceans, and fish [4]. Recently, the cultivation of microalgae as an alternative crop was proposed [5], as they can provide nutritious food for human. Furthermore, some algal microorganisms that can be used for producing antibiotics, toxins, pigments, plant growth regulators, and bioactive compounds were also discussed [6]. In terms of environmental protection, there are microalgae, including *Chlamydomonas reinhardtii* and *Chlorella vulgaris*, used for cleaning polluted water [7]. Bioethanol components in some microalgae, such as *Desmodesmus* sp., are employed to produce biodiesel [8] as up to 70% of those microalgae’s dry mass are hydrocarbons.

In practice, microalgae can be cultured in either raceways, open ponds, or closed cultivation systems, such as photobioreactors. While cultivating microalgal species in an open pond has a low cost and can provide flexible scalability, a closed system of culturing microalgae is more efficient in terms of controlling the growth rate and biomass productivity [1], which play important roles in cases where the microalgae are considered as commercial products. For example, a 40 liter closed system for culturing flagellates to concentrate the microalgal biomass was proposed in [9]. Two types of microalgae for larval fish, *Tetraselmis suecica and Brachionus plicatilis*, can be continuously cultivated in a customized cultivation system designed and built by Sananurak et al. in [10]. Naumann et al. developed a twin-layer solid-state bioreactor [11] for growing four types of microalgae, such as *Isochrysis* sp. *T.ISO*, *Tetraselmis suecica*, *Phaeodactylum tricornutum*, and *Nannochloropsis* sp., which can be utilized as live feeds in hatcheries. It is noted that a closed cultivation system allows growers to easily monitor crucial information of microalgae over time, which they can then employ to implement a closed loop control mechanism to automatically and efficiently operate all of the cultivation procedures [12]. For instance, one optimized up-scaled bioreactor for cultivating microalgal strain *S. platensis* was proposed in [13], where environmental parameters such as the pH condition, liquid level, and temperature during the culturing operations are remotely monitored through a smartphone app. Some discussions about employing advanced technologies including the Internet of Things or machine learning in intelligently farming microalgae were also presented in a recent work [14]. Nevertheless, one of the critical information normally required to be monitored in culturing microalgae, particularly in real time, is their density [6], since it allows growers to optimally control both nutrients and cultivating conditions.

The density of microalgae can be defined by the number of microalgal cells per mL [2,15]. In fact, an accurate number of algal cells can be obtained by carefully examining an aliquot through a microscope [16]. However, this process is tedious and time consuming, and particularly impractical in closed cultivation systems if an automatic control strategy is implemented. Thus, to efficiently and practically estimate the microalgal density, some estimation methods have been proposed [17]. For instance, the authors of the work [18] proposed to measure the oxygen levels of microalgae generated during their photosynthesis in a closed photobioreactor and analyze the data to indirectly estimate their density. Zhou et al. in [19] also relied on photosynthesis but exploited an in situ optical device to directly examine microalgae’s in vivo synthesis quantity to compute a density value. In a similar manner, the work [20] exploited another optical meter based on the spectrophotometric and fluorimetric principles to measure the turbidity of microalgae in a photobioreactor. The optical density indicator results were then used to calculate the concentration of those algal microorganisms.

Another class of methods proposed to monitor microalgal density is based on image processing. Thanks to low-cost camera sensors, image-based approaches are widely used [2,6,16,21,22,23,24]. Specifically, images of microalgae can be captured by a camera and then analyzed by an image processing technique, where extracted information can be utilized to calculate the density of microalgae presenting in the images. An advantage of these methods is that they are less invasive, nondestructive, and more biosecure. In the work [6], Jung et al. proposed to employ a camera sensor to digitally capture a top view of a microalgal photobioreactor and then analyze light distribution profiles in the captured images to indirectly quantify the cell density. Uyar in their work [22] developed a mechanism that relies only on a blue color channel of microalgal images to predict the algal cell concentration. In contrast, in our previous work [16], when taking *Chlorella vulgaris* microalgae into consideration, we discovered that the information carried by a blue channel in digitized images is insignificant. We then proposed to use only averages of red and green color channels of captured images for estimating the density of algal species. By considering all three red, green, and blue (RGB) color channels of captured images, Winata et al., in the work [24], first normalized them separately and then converted normalized RGB images to grayscale ones, where the microalgal concentration can be quantified.

So far, to the best of our knowledge, in most image-based microalgal density estimation methods, pixel values of measured images are averaged, which are then correlated with known density values in a regression model to estimate the concentration of microalgae in a new image. For instance, in [2,6,23,24], the authors, by exploiting different ratios of the red, green, and blue color components, converted RGB color images to grayscale ones. Each grayscale image was then averaged to a scalar value to input into a linear regression model. In our previous work [16], we averaged the red and green color channels of each measured RGB color image as inputs of a nonlinear Gaussian process model for prediction.

**Figure 1:**
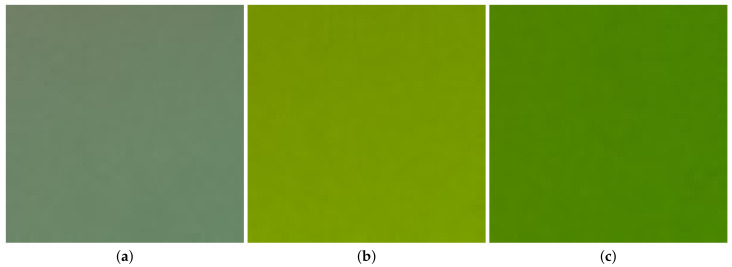
Examples of the cropped images of microalgae corresponding to density of (**a**) 5.08 million cells per mL, (**b**) 22.02 million cells per mL, and (**c**) 41.88 million cells per mL.

Since averaging pixel values in digitized images may not provide rich information of microalgae presenting in measured images, in this work, we investigated more advanced features from images as inputs of a regression model. As can be seen in the previous works [16,24] and in Figure 1 in the following discussion, since the microalgal image data are quite uniform, analyzing the textures of images is critical for understanding microalgal density information. In fact, the texture of an image is defined by the spatial relationship of pixel values or the variation in color intensity within the image [25]. Once the image texture is analyzed, information of the spatial distribution of pixel values in a given image can be quantified. Thus, in this work, we propose exploiting image texture characteristics as features to be input into a regression model for estimating the microalgal density. In addition to averages of pixel values, the image texture features considered in this work include confidence intervals of means of pixel values, powers of spatial frequencies presenting in images, and entropies accounting for pixel distribution. These diverse features can provide more information of the microalgae density, which can lead to more accurate estimation results.

On the other hand, since the relationship between the image features and microalgal densities can be linear or nonlinear, we propose to exploit an L1-regularization-based approach called least absolute shrinkage and selection operator (LASSO) [26,27] as a regression model. Fundamentally, regularization can help a regression model avoid the over-fitting of data [28]. Furthermore, coefficients of the features in the model can be optimized in a way that prefers the more informative features, which results in a well-fit model to the data. Eventually, the microalgal density can be more accurately estimated. In order to evaluate the effectiveness of the proposed approach, we implemented it in a dataset collected by a sensing system [29] monitoring the *Chlorella vulgaris* microalgae strain. The obtained results demonstrate the outperformance of the proposed method compared with several existing methods.

The remainder of the paper is arranged as follows. Section 2 presents how a dataset of the *Chlorella vulgaris* microalgae can be gathered by using our camera-based algae monitoring system [16]. In Section 3, we discuss how to design features of microalgal images by analyzing their textures. The LASSO-based regression model is introduced in Section 4, where a model for estimating the density of microalgae given their image features is derived. We then extensively discuss possible combinations of image features as inputs and corresponding results as outputs of the estimation model in Section 5, where the effectiveness of the proposed approach is verified in real-world experiments. Some conclusions of the discussion are drawn in Section 6.

## 2. Data Collection

In order to investigate how image texture features can be exploited to estimate the density of microalgae, we developed a new monitoring system that is non-destructive and biosecure since it only captures images of microalgae from a distance. In this section, we introduce the monitoring system and its captured image data, which are then utilized to interpret the density information.

### 2.1. Monitoring System

**Figure 2:**
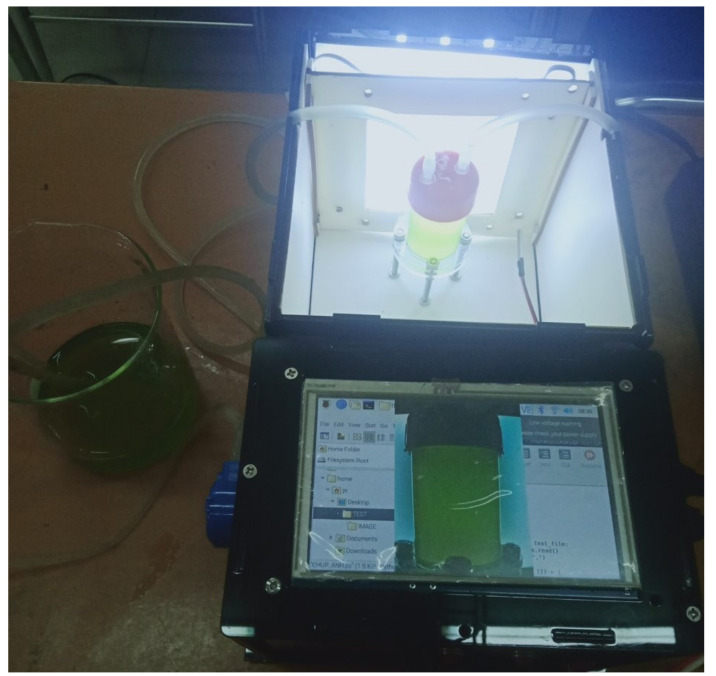
The low-cost system for capturing images of microalgae.

With the expectation that the proposed estimation framework can be widely implemented in practice, a low-cost microalgae monitoring system was developed. The premise behind the monitoring system is that the microalgae solution is slowly and continuously pumped from a cultivation tank into a sample container and then refluxed back to the tank. The sample container is transparent and located in a dark box. A low-cost Raspberry Pi camera was deployed on a wall of the box to take photos of the sample container. Due to the transparency of the container, the captured images present information of microalgae. It is noted that, for the photography in the dark box, an artificial light was generated. Using the artificial light aims to eliminate any disturbances caused by uncertain natural light, which guarantees the best quality of the captured images. Since the sensing devices do not destruct the microalgae structure, the monitoring system is non-invasive and biosecure, which differs from some invasive techniques that include the dry cell weight [30]. Furthermore, the system can provide continuous monitoring over time without disturbing the growth of microalgae. The microalgae monitoring system in a working state is demonstrated in Figure 2. The development of the system cost approximately USD 150. For more details of this sensing system, interested readers are referred to our previous work [16].

**Figure 3:**
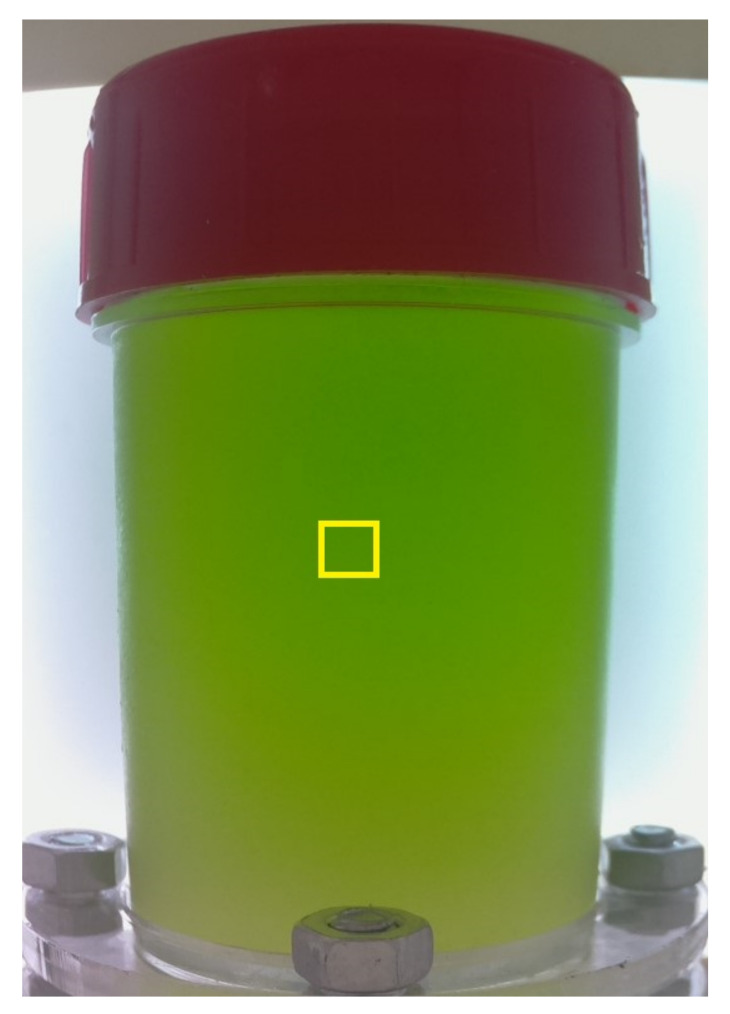
A raw image of microalgae captured by our developed system.

To demonstrate the study in this work, we used the monitoring system to collect the image data of one specific microalgae strain called *Chlorella vulgaris*. A captured image example at a resolution of 800×600 is illustrated in Figure 3. Moreover, to verify our proposed approach using LASSO, we gathered multiple images of *Chlorella vulgaris* microalgae at different densities. In particular, we started with a microalgal solution of 42.3 million cells per mL, where one image of the microalgae presented in the solution was taken. We then repeatedly diluted the solution by pure water 128 times and, at each new solution generated, a corresponding image of microalgae was captured. There are a total of 129 images of microalgae presented in 129 samples with different densities.

On the other hand, we ultimately aim to efficiently estimate the density of microalgae given their image; that is, the proposed approach cannot be truly verified without knowing the ground truth densities in those solution samples. Therefore, we manually counted microalgae cells in each representative sample of 129 solutions through a time-consuming technique called the direct microscopic count method. Details of how we obtained the ground truth of 129 densities of microalgae presented in the 129 corresponding images can be found in [16]. It is noted that, in the verification process of our proposed approach, we estimated the density of microalgae by using captured image data. The estimation results were then compared with their corresponding ground truth densities to assess the accuracy of the estimation method.

### 2.2. Preprocessing Data

As can be seen in Figure 3, a raw image of microalgae captured by the monitoring system contains not only microalgae information but also some background. Apparently, a more expensive camera can help focus on the microalgae region. Unfortunately, the low-cost Raspberry Pi OV 5647 camera sensor used in the monitoring system does not have zoom functionality. On the other hand, analyzing this whole raw image is highly computationally expensive. In fact, it is not necessary to analyze the entire raw image since our proposed approach employs the learning principle. Therefore, we propose to crop the raw image to a sub-image at a resolution of 50×50, whose top-left coordinate is at (375,275) in the original image. The location of the sub-image in the original image is illustrated by a yellow square in Figure 3. In the proposed method, we solely use the information in the cropped region to input into a model and train the model parameters based on that information only. In the implementation, given a new raw image, we then take only the information in the same cropped region to input into the trained model for the estimation. Information outside the yellow square is not considered in the learning model. Three examples of the cropped images corresponding to different densities are demonstrated in Figure 1. The problem now becomes analyzing a cropped image (e.g., as demonstrated in Figure 1) to estimate the microalgal density.

**Figure 4:**
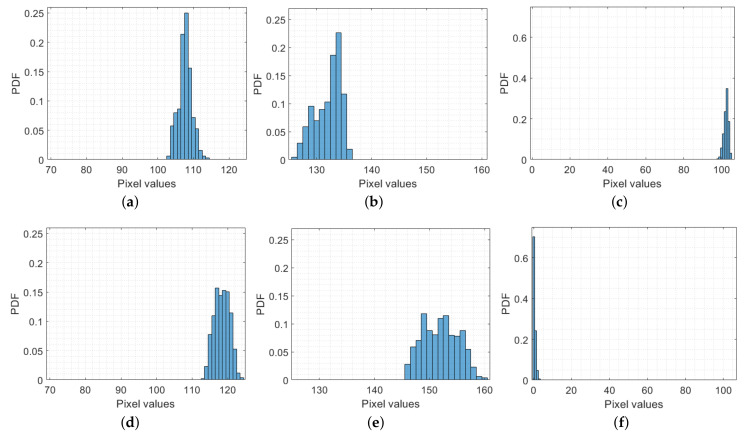

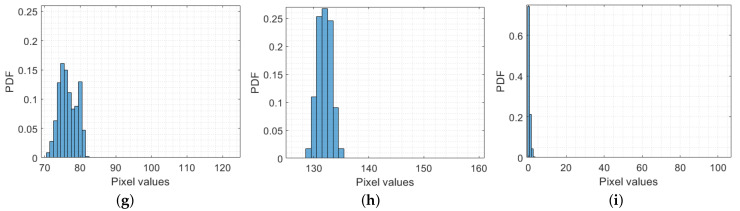
Distributions of the pixel values in color channels of the cropped images of Figure 1a (first row), Figure 1b (second row), and Figure 1c (last row), respectively. (**a**) Red channel of Figure 1a; (**b**) green channel of Figure 1a; (**c**) blue channel of Figure 1a; (**d**) red channel of Figure 1b; (**e**) green channel of Figure 1b; (**f**) blue channel of Figure 1b; (**g**) red channel of Figure 1c; (**h**) green channel of Figure 1c; (**i**) blue channel of Figure 1c.

It is noted that each cropped image has three color channels: red, green, and blue. Intuitively, the distribution of microalgae in the image looks quite uniform. Nevertheless, to learn the characteristics of the image, we analyzed its color channels separately to see how the pixel values are distributed. In particular, the pixel values in each color channel were grouped into a histogram, where their distribution can be statistically evaluated. For instance, nine histograms of nine color channels in three cropped images in Figure 1 are demonstrated in Figure 4, respectively. Each row of Figure 4 demonstrates three color channels of one cropped image whereas each column illustrates one color (left—red, middle—green, and right—blue). For comparison, each color distribution is plotted in the same scale. Overall, the distribution range of the pixel values in each color channel is quite concentrated. More specifically, the standard deviation of the pixel values in these nine color channels ranges from 0.6 to 3.2, which accounts for a pretty uniform distribution of microalgae in the captured images as can be seen in Figure 1. The next question is what features can be established in the data for the purpose of estimating the microalgal density, which are discussed in the following section.

## 3. Feature Design

Since the image data are quite uniform, analyzing the texture of images is critical for understanding microalgal density information. The texture of an image is defined by the spatial relationship of pixel values or the variation in color intensity within the image [25]. In other words, texture provides information of the spatial distribution of pixel values in a given image.

Given the examples of distributions of pixel values in the color channels as illustrated in Figure 4, it can be observed that, though the distribution in each color channel is quite uniform, the distribution range is different from one image to another. Hence, we extracted features from the original color image or each color channel, which can be used in the LASSO model as discussed in the next section for estimating the density of microalgae. In the following subsection, we will discuss some typical mechanisms employed to characterize features of each color channel in the cropped image.

### 3.1. Averages of Color Channels

Due to the uniformity of the pixel values in each color channel as illustrated in Figure 4, some existing works proposed utilizing the average of pixel values in a color channel as a predictor feature in building an estimation model. For instance, in [6,24], the authors used a formula proposed by the International Telecommunication Union (ITU) to compute an average value of a grayscale image as follows:

(1) $$GS_{ITU}=0.222R+0.707G+0.071B,$$

where $GS_{ITU}$ is a grayscale average and *R*, *G*, and *B* are the average values of the red, green, and blue color channels, respectively. Likewise, Córdoba-Matson et al. in [2] proposed two other formulae to calculate a grayscale average from *R*, *G*, and *B*, which are specified by

(2) GS1=0.2989R+0.5870G+0.1140B,

and

(3) GS2=0.333R+0.333G+0.333B.

The idea in [2,6,24] is to feature a captured image of microalgae by a grayscale average value, which is then linearly correlated with the density of microalgae by a linear model. The trained linear model can be used to predict the microalgal density in a newly captured image.

**Figure 5:**
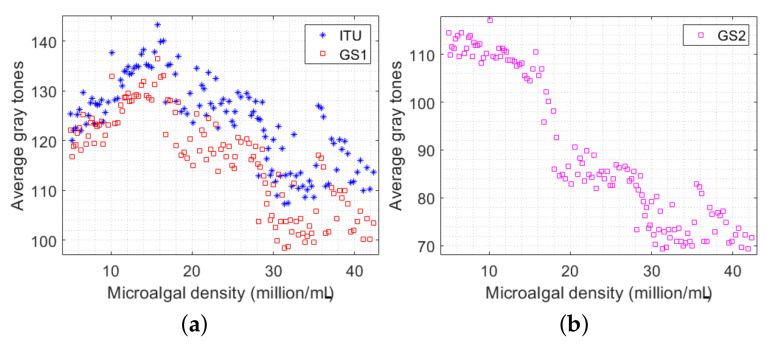
Grayscale average values of the microalgal images versus their corresponding densities, where the gray tones are converted based on (**a**) ITU [6,24] and GS1 [2] criteria and (**b**) GS2 [2] criterion.

Though the linear models based on ITU, GS1, and GS2 were verified in some microalgal strains, including *Synechococcus* sp., *Desmodesmus* sp., *Scenedesmus* sp., *Dictyosphaerium* sp., *Isochrysis galbana*, and *Klebsormidium* sp., they do not fit well to our dataset collected from the *Chlorella vulgaris* microalgae strain. More specifically, we implemented (1)–(3) with our image dataset and plotted the resulting grayscale average values against the corresponding densities. The visualization results are demonstrated in Figure 5. It can be clearly seen that relationships between the microalgal densities and the grayscale average values obtained by the ITU and GS1-based methods are highly nonlinear, whereas that of the results obtained by the GS2 based algorithm is slightly linear. In other words, these linear models do not work well in a generic scenario for any type of microalgal strain.

In contrast, in our previous work [16], we proposed to only utilize the average values of the red and green color channels of an image as the input of a two-dimensional (2D) nonlinear model, whose output is a corresponding density of microalgae presenting in the image. Using Gaussian processes, we then developed a framework to predict the microalgal density from an image.

In this work, we propose to exploit the average values of all three color channels in each captured image as three features presenting information of microalgae in the image. It is noted that the median can be used in place of the average, particularly in scenarios where outliers in the data are inevitable.

### 3.2. Mean Intervals of Color Channels

Since a single average value does not provide information of the distribution range of pixel values, we then propose to exploit the confidence interval of the average value. Given a confidence level, we can probabilistically estimate the distribution range of the average value [31]. Let us take a red color channel as an example: the confidence interval μ of the average of pixel values in the channel can be computed by (4) as follows:

(4) $$R-z_{\alpha /2}\frac{\sigma }{\sqrt{N_{px}}}\leq \mu \leq R+z_{\alpha /2}\frac{\sigma }{\sqrt{N_{px}}},$$

where *R* is the average value of the red channel, σ is the standard deviation of the pixel values, $N_{px}$ is the number of pixels, $z_{\alpha /2}$ is the *z*-value, and α is the significant level. If a 95% level of confidence is used, then $z_{\alpha /2}=1.96$.

Therefore, instead of the average value, we propose to utilize two ends of the confidence interval as two features of a color channel. In other words, we define two interval features for a red color channel as $R_{L}=R-z_{\alpha /2}\frac{\sigma }{\sqrt{N_{px}}}$ and $R_{R}=R+z_{\alpha /2}\frac{\sigma }{\sqrt{N_{px}}}$. Likewise, $G_{L}$, $G_{R}$, $B_{L}$, and $B_{R}$ can be computed for the interval features of the green and blue color channels. Overall, we have six interval features for each color image of microalgae.

### 3.3. Spatial Frequency

In the signal context, the texture of an image can be represented by the content of its local spectrum or frequency; that is, variations in the texture in a region of an image can cause changes in local spatial frequencies [25]. Given this principle, we propose to compute the power spectrum of each color channel of microalgae images. More specifically, 2D discrete Fourier transform (DFT) [32] can be calculated by (5) as follows:

(5) $$F_{x,y}\left(u,v\right)=\overset{M-1}{\underset{m=0}{\sum }}\overset{N-1}{\underset{n=0}{\sum }}e^{-j2\pi um/M}e^{-j2\pi vn/N}f_{\hat{x},\hat{y}},$$

where $\hat{x}=x-M+m+1$, $\hat{y}=y-N+n+1$. $f_{\hat{x},\hat{y}}$ is the ${\left(x,y\right)}^{th}$ pixel in a color channel at a resolution of M×N. *u* and *v* are spatial frequencies and $F_{x,y}\left(u,v\right)$ is the ${\left(u,v\right)}^{th}$ bin of the DFT result.

**Figure 6:**
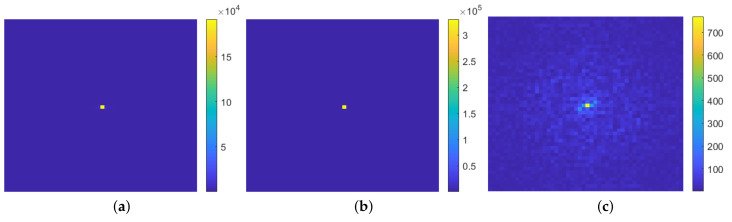
Power spectrum of spatial frequencies in three color channels of the microalgae image shown in Figure 1c. (**a**) Red channel; (**b**) green channel; (**c**) blue channel.

We implemented the spatial frequency technique in our dataset, and, as an example, the power spectra of three color channels of the image as demonstrated in Figure 1c are illustrated in Figure 6. As can be seen in Figure 4g–i, since the distribution range of pixel values in each color channel is different, the corresponding power spectra in Figure 6 are also different. Therefore, we propose to form three spatial frequency features in each microalgae image by computing average values of three corresponding power spectra, which are called $R_{FT}$, $G_{FT}$, and $B_{FT}$.

### 3.4. Entropy

In texture analysis, another measure for characterizing the texture of an image is entropy [33]. Theoretically, entropy is used to measure the randomness or information content of the pixel distribution. It has been used as a feature in texture classification [34]. In this work, we propose to calculate the entropies of the color channels in each image of microalgae and employ the entropy results as features to present characteristics of the image. Mathematically, the entropy of each color channel [35] can be specified by (6) as follows:

(6) $$R_{entropy}=-\overset{K}{\underset{k=1}{\sum }}p_{k}\log_{2}\left(p_{k}\right),$$

where $R_{entropy}$ is entropy of the red color channel and $p_{k}$ is the probability of the $k^{th}$ pixel value. In the implementation, we simply created a histogram of all pixel values in each color channel, with a bin width of 1, as can be seen in Figure 4. Then, $p_{k}$ can be considered as a normalized histogram count from the histogram, and *K* is the number of histogram bins. Eventually, for each color image of microalgae, we can compute three entropy features, including $R_{entropy}$, $G_{entropy}$, and $B_{entropy}$, which can be employed in further analysis in estimating the microalgal density.

## 4. L1-Regularization Based Estimation Method

We have now learned that there are several methods for extracting features from images of microalgae as discussed in Section 3. It is noted that we can even formulate new features from the existing features that are characterized directly from an image. For instance, given the features we discussed in Section 3, new features such as $R_{entropy}^{2}$ or $R_{entropy}\times G_{entropy}$ can be obtained. Thus, the microalgal density estimation problem becomes building the relationship between the image features and density values. In other words, if a model between the image features and density values can be built, it can be employed to predict the density of microalgae given their image.

It is assumed that there are *q* features $f_{1}$, $f_{2}$, ⋯, $f_{q}$ extracted from an image of microalgae that have a density of *d*. Let us propose a linear relationship between the image features and density values, specified by (7) as follows:

(7) $$d=\beta_{0}+\beta_{1}f_{1}+\beta_{2}f_{2}+\cdots +\beta_{q}f_{q},$$

where $\beta_{j}$ is an unknown coefficient that can be estimated through training data. We assume that there are *n* collected images of microalgae and *n* corresponding density values $d_{1}$, $d_{2}$, ⋯, $d_{n}$, which leads to *n* sets of features ${\left\{f_{i1},f_{i2},\cdots ,f_{iq}\right\}}_{i=1}^{n}$. Then, the coefficients $\beta_{1},\;\beta_{2},\;\cdots ,\;\beta_{q}$ can be straightforwardly calculated by the use of the least square algorithm [26].

However, due to the complexity of the dataset, the least square technique may cause under-fitting or over-fitting problems [26], which leads to inaccuracy in the prediction. Therefore, in this work, we propose to employ an L1-regularization-based approach called least absolute shrinkage and selection operator (LASSO) [26,27] to optimally estimate the coefficients $\beta_{1},\;\beta_{2},\;\cdots ,\;\beta_{q}$. Fundamentally, regularization can help a regression model to avoid the over-fitting of data [28].

### 4.1. LASSO

The premise behind LASSO is that L1 regularization adds a penalty equal to absolute values of the coefficients $\beta ={\left[\beta_{1},\beta_{2},\cdots ,\beta_{q}\right]}^{T}$. More specifically, the coefficients β can be optimized by [26,36]

(8) $$\hat{\beta }={\mathrm{argmin}}_{\beta }\left[\frac{1}{2}\overset{n}{\underset{i=1}{\sum }}{\left(d_{i}-\beta_{0}-\overset{q}{\underset{j=1}{\sum }}f_{ij}\beta_{j}\right)}^{2}+\lambda \overset{q}{\underset{j=1}{\sum }}\left|\beta_{j}\right|\right],$$

where λ is a penalty term that can be predefined.

It is noted that the regularization in (8) can lead to a sparse model. In other words, some coefficients obtained by (8) may become less important than the others and become zeros. The importance of a coefficient is measured by its absolute value. In scenarios when some coefficients become zeros, their corresponding features are eliminated from the model, which results in a reduction in the computational complexity. This attribute of the regularization provides the LASSO model with the ability to significantly rely on more important features, which eventually leads to a more accurate prediction in regression.

### 4.2. Penalty Term Learning

**Figure 7:**
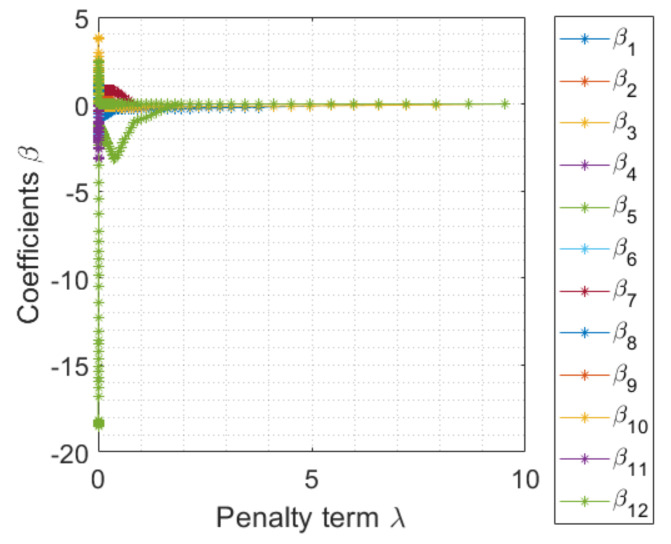
An example of the coefficients $\hat{\beta }$ given the different penalty term λ values.

It can be seen in (8) that, when the penalty term λ is zero, the optimization becomes the least square. In contrast, when λ tends to infinity, all of the coefficients $\hat{\beta }$ approach zero, which makes the regression model well under-fitting. To demonstrate the influence of choosing λ on the resulting coefficients $\hat{\beta }$, we conducted an experiment in our dataset, where we defined 12 features, such as *R*, *G*, *B*, $R_{entropy}$, $G_{entropy}$, $B_{entropy}$, $R_{entropy}^{2}$, $G_{entropy}^{2}$, $B_{entropy}^{2}$, $R_{entropy}\times G_{entropy}$, $R_{entropy}\times B_{entropy}$, and $G_{entropy}\times B_{entropy}$, which were extracted from the image data. In this experimental example, we set λ to 100 values ranging from 0 to 10. After optimizing (8), the obtained coefficient results are depicted in Figure 7. The results verify the argument that, when λ increases, all of the coefficients converge to zero. It is noted that, when λ is small, some resulting coefficients are very close to each other; that is, their corresponding features contribute quite similarly to the model.

**Figure 8:**
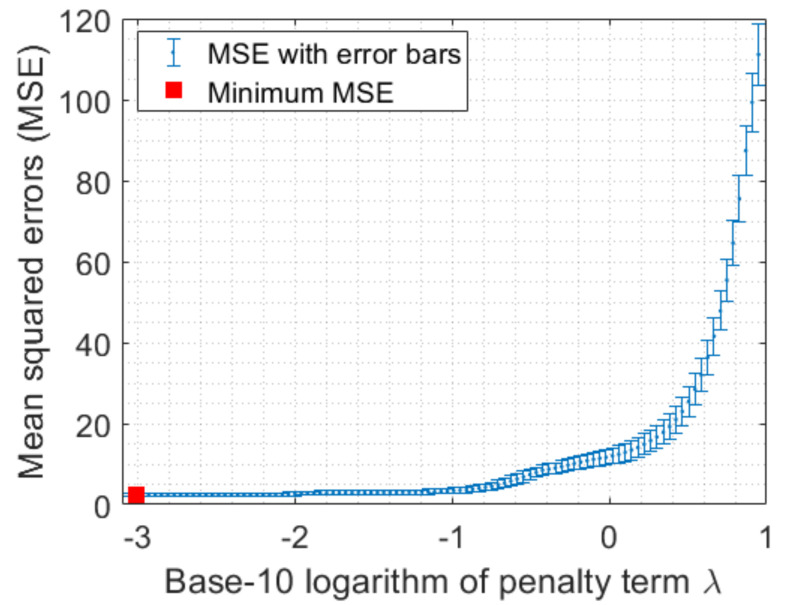
The mean square errors obtained by the *k*-fold cross-validation algorithm given the different penalty term λ values.

Given the example illustrated in Figure 7, we now investigate what value we should use for the penalty term λ. In order to automatically identify the best value for λ given the dataset, we propose to exploit the *k*-fold cross-validation technique [37]. For instance, one can set λ to one of 100 values ranging from 0 to 10. For each λ value, it runs the *k*-fold cross-validation algorithm in the given dataset, where, at each iteration, the optimization (8) is solved. A mean of squared errors (MSE) or cross-validated squared residuals over *k* folds can also be calculated. After running all possible values of λ, the best penalty term is selected by the corresponding minimum MSE. As a demonstration, we implemented this learning technique in our dataset with 12 aforementioned features, in which, we chose k=5. The obtained MSE results with error bars are depicted in Figure 8, where the minimum MSE is highlighted by a red square, which corresponds to the selected λ.

The next argument is how many folds in the cross-validation method should be partitioned from the original dataset? We empirically implemented the technique learning λ in our image data with three different *k* values. The obtained results demonstrate that the optimal penalty term is consistent regardless of *k*, as summarized in Table 1.

### 4.3. Microalgal Density Estimation

Once the penalty term λ is determined, given training data, the coefficients $\hat{\beta }$ can be calculated by optimizing (8); that is, a model of the microalgal density is learned. In the prediction step, when a new image of microalgae is taken, *q* features presenting for the microalgae information can be extracted from the image and input into the learned model. The corresponding density of the microalgae can be estimated by (9) as follows.

(9) $$\hat{d}={\hat{\beta }}_{0}+{\hat{\beta }}_{1}f_{1}+{\hat{\beta }}_{2}f_{2}+\cdots +{\hat{\beta }}_{q}f_{q},$$

where $\hat{\beta }=\left[{\hat{\beta }}_{0},\;{\hat{\beta }}_{1},\;{\hat{\beta }}_{2},\;\cdots ,\;{\hat{\beta }}_{q}\right]$, and $\hat{d}$ is the estimated density.

## 5. Experimental Result Analysis

Given the LASSO estimation approach, we now discuss how to apply the features introduced in Section 3 to efficiently estimate the density of microalgae given their images. Since there are multiple features being extracted from an image, multiple scenarios of combining different features can be investigated. It is noticed that not all features can provide good information of microalgae: different combinations of different features may lead to different estimation results. Therefore, in this work, we consider the typical combinations from four sets of the features presented in Section 3 and analyze the results to derive the best combination of the features, which can lead to the most accurate estimation of microalgal density, at least with our dataset. The procedure can be easily extended to any other microalgae datasets.

**Figure 9:**
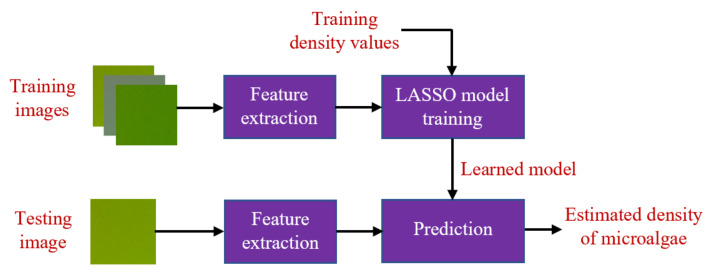
Framework of the validation procedure.

In order to validate the estimation accuracy in each scenario, we randomly partitioned our 129 data samples, including 129 color images and 129 ground truth density values, into two subsets. The first subset of 100 samples was used for training, and the second subset of 29 samples was used for testing. The validation framework is summarized in Figure 9. Since selecting training and testing data in each validation is random, to statistically verify the effectiveness of the proposed approach, in each scenario, we repeated the implementation 1000 times. At each run, we computed a root mean square error (RMSE) between the estimated density results and the ground truth in the testing subset. The 1000 root mean square errors (RMSEs) in each scenario were then condensed into two statistical parameters of mean and standard deviation (std) for comparisons among the combination scenarios.

### 5.1. Single Feature Combinations

In the first evaluation, we consider four scenarios where each scenario contains a single type of feature as discussed in Section 3. The combinations are as follows:

- S1: Features of average values of all three color channels: *R*, *G* and *B*.
- S2: Six interval features: $R_{L}$, $R_{R}$, $G_{L}$, $G_{R}$, $B_{L}$, and $B_{R}$.
- S3: Three spatial frequency features: $R_{FT}$, $G_{FT}$, and $B_{FT}$.
- S4: Three entropy features: $R_{entropy}$, $G_{entropy}$, and $B_{entropy}$.

The evaluation results presented by the mean and std values obtained in these four scenarios are summarized in Table 2.

It can be seen from Table 2 that, in the first three scenarios, the estimation results are quite comparable. Nevertheless, in the fourth scenario, the estimated density results are highly inaccurate. It seems that the entropy features do not carry much information about microalgae in the images.

### 5.2. Two-Feature Combinations

If a single type of feature does not provide a good estimation of the microalgal density, combining two types of features in a learning model may enrich the information of microalgae, which can lead to better prediction results. There are six possible combinations of two types of features from four sets of the features that we discuss in this work. Let us examine those scenarios as follows:

- S5: Features of three color channel averages and three entropies: *R*, *G*, *B*, $R_{entropy}$, $G_{entropy}$, and $B_{entropy}$.
- S6: Features of six interval bounds and three entropies: $R_{L}$, $R_{R}$, $G_{L}$, $G_{R}$, $B_{L}$, $B_{R}$, $R_{entropy}$, $G_{entropy}$, and $B_{entropy}$.
- S7: Features of three color channel averages and three spatial frequency powers: *R*, *G*, *B*, $R_{FT}$, $G_{FT}$, and $B_{FT}$.
- S8: Features of three color channel averages and six interval bounds: *R*, *G*, *B*, $R_{L}$, $R_{R}$, $G_{L}$, $G_{R}$, $B_{L}$, and $B_{R}$.
- S9: Features of three spatial frequency powers and three entropies: $R_{FT}$, $G_{FT}$, $B_{FT}$, $R_{entropy}$, $G_{entropy}$, and $B_{entropy}$.
- S10: Features of six interval bounds and three spatial frequency powers: $R_{L}$, $R_{R}$, $G_{L}$, $G_{R}$, $B_{L}$, $B_{R}$, $R_{FT}$, $G_{FT}$, and $B_{FT}$.

The validation results in these six scenarios of two types of the features combinations are tabulated in Table 3. Overall, the estimated results obtained by combining two types of the features are better than those obtained by combining a single type of the features. On the other hand, whenever the entropy appears in the feature sets, the prediction results are slightly better than the others; that is, under mixture with other features, the entropy can provide rich information of the microalgae.

### 5.3. Three and Four-Feature Combinations

We now consider scenarios of combining three or four types of features to see whether a greater variety in the types of image features involved in one learning process can result in a more accurate prediction of the microalgal density. There are five scenarios when combining three or four types of image texture features, which are as follows:

- S11: Features of three color channel averages, six interval bounds, and three entropies: *R*, *G*, *B*, $R_{L}$, $R_{R}$, $G_{L}$, $G_{R}$, $B_{L}$, $B_{R}$, $R_{entropy}$, $G_{entropy}$, and $B_{entropy}$.
- S12: Features of three color channel averages, six interval bounds, and three spatial frequency powers: *R*, *G*, *B*, $R_{L}$, $R_{R}$, $G_{L}$, $G_{R}$, $B_{L}$, $B_{R}$, $R_{FT}$, $G_{FT}$, and $B_{FT}$.
- S13: Features of three color channel averages, three entropies, and three spatial frequency powers: *R*, *G*, *B*, $R_{entropy}$, $G_{entropy}$, $B_{entropy}$, $R_{FT}$, $G_{FT}$, and $B_{FT}$.
- S14: Features of six interval bounds, three entropies, and three spatial frequency powers: $R_{L}$, $R_{R}$, $G_{L}$, $G_{R}$, $B_{L}$, $B_{R}$, $R_{entropy}$, $G_{entropy}$, $B_{entropy}$, $R_{FT}$, $G_{FT}$, and $B_{FT}$.
- S15: All four types of the features as discussed in Section 3: *R*, *G*, *B*, $R_{L}$, $R_{R}$, $G_{L}$, $G_{R}$, $B_{L}$, $B_{R}$, $R_{FT}$, $G_{FT}$, $B_{FT}$, $R_{entropy}$, $G_{entropy}$, and $B_{entropy}$.

Similar to the previous considerations, the mean and std results obtained in these five scenarios are also summarized in Table 4 for comparison. It can be seen that combining more than two types of image features for estimating the microalgal density does not enhance the estimation results compared with those in the cases where two types of features are used, as demonstrated in Table 3. Therefore, we propose to employ only two types of features in the training and prediction processes to reduce the computational complexity.

### 5.4. Higher-Order and Nonlinear Entropy Features

We now propose to create new features from the existing ones extracted from images, e.g., $R^{2}$ or $R_{entropy}\times G_{entropy}$, and incorporate them into the input of the training data. Manipulating the existing features to higher-order or nonlinear features may weigh the LASSO model in a different manner, which can lead to a better estimation. Out of the four types of features, we picked the entropy for the manipulation. Though the entropy features, when standing alone, do not provide good estimation results, as demonstrated in Section 5.1, when combined with other types of features, they could lead to an improvement in the estimation accuracy as discussed in Section 5.2. By using the higher-order and nonlinear entropy features, we generated six combination scenarios for discussion as follows:

- S16: Features including *R*, *G*, *B*, $R_{entropy}$, $G_{entropy}$, $B_{entropy}$, $R^{2}$, $G^{2}$, $B^{2}$, $R_{entropy}^{2}$, $G_{entropy}^{2}$, and $B_{entropy}^{2}$.
- S17: Features including *R*, *G*, *B*, $R_{entropy}$, $G_{entropy}$, $B_{entropy}$, $R_{entropy}^{2}$, $G_{entropy}^{2}$, and $B_{entropy}^{2}$.
- S18: Features including $R_{entropy}$, $G_{entropy}$, $B_{entropy}$, $R_{entropy}^{2}$, $G_{entropy}^{2}$, and $B_{entropy}^{2}$.
- S19: Features including *R*, *G*, *B*, $R_{entropy}$, $G_{entropy}$, $B_{entropy}$, $R_{entropy}^{2}$, $G_{entropy}^{2}$, $B_{entropy}^{2}$, $R_{entropy}^{3}$, $G_{entropy}^{3}$, and $B_{entropy}^{3}$.
- S20: Features including *R*, *G*, *B*, $R_{entropy}$, $G_{entropy}$, $B_{entropy}$, $R_{entropy}^{2}$, $G_{entropy}^{2}$, $B_{entropy}^{2}$, $R_{entropy}\times G_{entropy}$, $R_{entropy}\times B_{entropy}$, and $G_{entropy}\times B_{entropy}$.
- S21: Features including *R*, *G*, *B*, $R_{entropy}$, $G_{entropy}$, $B_{entropy}$, $R_{entropy}^{2}$, $G_{entropy}^{2}$, $B_{entropy}^{2}$, $R_{entropy}\times G_{entropy}$, $R_{entropy}\times B_{entropy}$, $G_{entropy}\times B_{entropy}$, and $R_{entropy}\times G_{entropy}\times B_{entropy}$.

After running six combination scenarios using the high-order and nonlinear features, the obtained results are tabulated in Table 5 for further analysis. Compared with the results in the previous discussion, the estimation results in five out of these six scenarios are more accurate.

In order to demonstrate why the entropy is used to create high-order and nonlinear features, in scenario S16, we exploited the second order of both the color channel averages and entropies ($R^{2}$, $G^{2}$, $B^{2}$, $R_{entropy}^{2}$, $G_{entropy}^{2}$, and $B_{entropy}^{2}$) in the LASSO model. However, in scenario S17, we dropped the features $R^{2}$, $G^{2}$, and $B^{2}$. The results in both of the scenarios that can be found in Table 5 are consistent; that is, the features $R^{2}$, $G^{2}$, and $B^{2}$ do not add more information of microalgae to the model. To reduce the computational complexity, we will not incorporate $R^{2}$, $G^{2}$, and $B^{2}$ in further consideration.

In scenario S18, we even dropped all of the features relating to the color channel averages. As can be seen in Table 5, the obtained results get worse. In other words, the features *R*, *G*, and *B* should remain in the training dataset. We then extended the entropy features to the third order in scenario S19. Nonetheless, the results are not better than those in S17, which empirically proves that the third-order features are insignificant.

Moreover, we considered a multiplication interaction between two features. For instance, in scenario S20, we employed three new features created by the entropies, including $R_{entropy}\times G_{entropy}$, $R_{entropy}\times B_{entropy}$, and $G_{entropy}\times B_{entropy}$. The prediction results obtained in Table 5 show a significant improvement in the accuracy compared with all of the others. We then added the multiplication interaction among three features, such as $R_{entropy}\times G_{entropy}\times B_{entropy}$ in scenario S21. Nonetheless, the obtained results were not enhanced compared with those in S20, though the computation was more complicated. Hence, we accepted scenario S20 as it can provide an acceptable estimation accuracy in our application [16].

It is noted that the procedure that we discuss in this section can be easily extended to other microalgal density estimation applications, where new features can be created and combined with others as the input of the LASSO model to ameliorate the estimation accuracy to an expected level. However, the trade-off between the accuracy and computational complexity should be practically considered.

### 5.5. Estimation Results

We now take the features combined in scenario S20 as the input of the LASSO model and examine the performance of the proposed approach in our dataset, along with that of the others, including the Gaussian process (GP)-based algorithm [16] and GS2-based technique [2].

**Figure 10:**
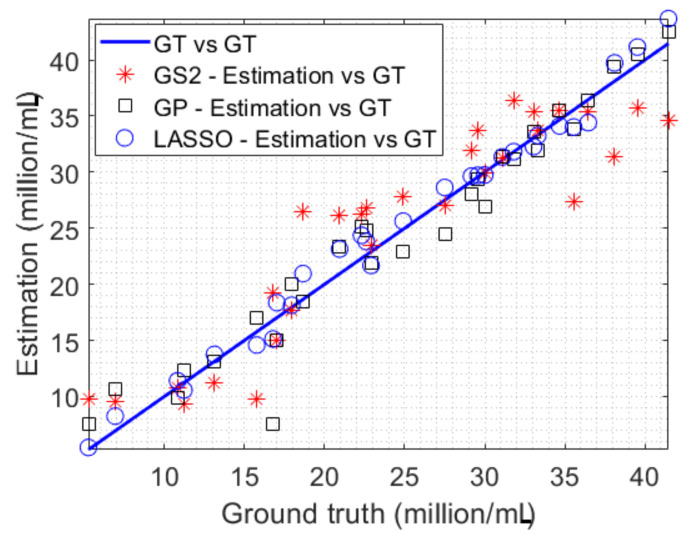
The microalgal density estimation results versus the ground truth in the testing data.

Let us consider one example of the training and testing data sets, where 100 image samples and 100 ground truth (GT) values of microalgal density in the training data were utilized to train three models including LASSO, GP and GS2. Given 29 images in the testing data, the trained models were then employ to estimate densities of the corresponding microalgae. The estimation results are compared with the GT in the testing data, as demonstrated in Figure 10. In an ideal case of absolutely accurate estimation, the estimation results should lie on the GT line (e.g., the blue line in Figure 10). In other words, if the estimated density values stay further from the GT line, the estimation method is less accurate. As can be seen in Figure 10, the proposed LASSO approach outperforms both the GP and GS2-based algorithms in the example.

**Figure 11:**
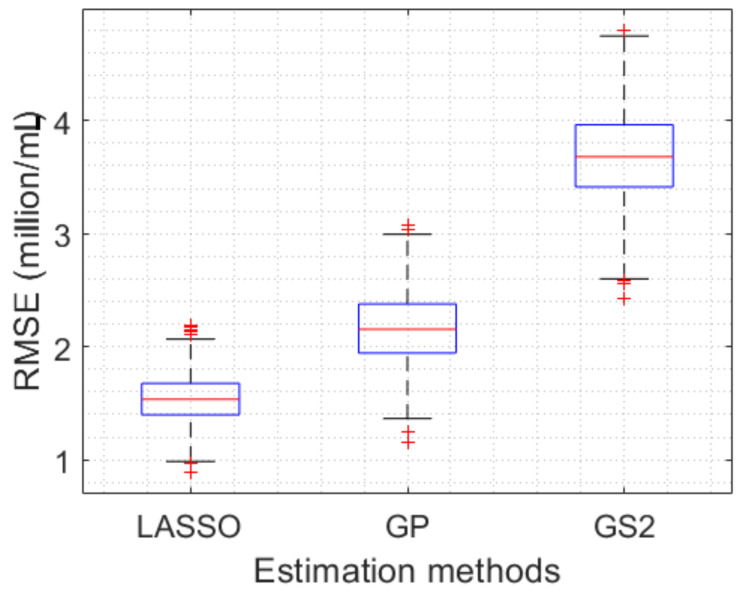
Boxplots of RMSEs obtained by the proposed approach compared with those obtained by GP [16] and GS2 [2].

In order to statistically conclude the outperformance of the LASSO estimation method, we repeated the implementation of three techniques 1000 times, where, in each implementation, the training and testing data sets were randomly selected. RMSEs between the estimated density results and the GT in the corresponding testing data set were also computed in 1000 implementations. The RMSEs results obtained by three methods are summarized by boxplots as illustrated in Figure 11. It can be clearly seen that the results obtained by the LASSO approach are more accurate than those obtained by the GP and GS2 techniques. More specifically, the mean and interquartile range of the estimation RMSEs in the LASSO implementations are 1.54 and 0.28, whereas those in the GP and GS2 experiments are 2.16 and 0.43, and 3.68 and 0.55, respectively.

## 6. Conclusions

This paper has discussed an image-based approach for efficiently estimating the microalgal density. The exploitation of some features of image texture, including color channel averages, confidence intervals of means of pixel values, powers of spatial frequencies, and entropies, has been proposed to represent characteristics of microalgae presenting in measured images. More importantly, a LASSO regression model was utilized to optimally select the most informative features, which leads to the best fitting to the data and more accurately estimated results of the microalgal density. The proposed approach was evaluated in the real-life dataset of monitoring the *Chlorella vulgaris* microalgae strain. The obtained results show that the average estimation error in the proposed method is approximately 1.54, whereas those in the state-of-the-art methods are 2.16 and 3.68, respectively.

By using the approach proposed in this work, we developed a monitoring system with a software-based human–machine interface to effectively monitor the microalgal density. The details of the applications and software can be found in [29].

## Figures and Tables

**Table 1:** The optimal penalty term at different *k* in the *Chlorella vulgaris* microalgae dataset.

	k=5	k=10	k=20
Minimum MSE	2.313	2.215	2.278
λ	0.001	0.001	0.001

**Table 2:** Comparison of estimation accuracy in the first four examined scenarios in Section 5.1.

	S1	S2	S3	S4
mean	2.40	2.12	2.36	4.90
std	0.25	0.26	0.28	0.67

**Table 3:** Comparison of estimation accuracy in the next six examined scenarios in Section 5.2.

	S5	S6	S7	S8	S9	S10
mean	1.75	1.78	1.89	2.09	1.79	2.09
std	0.23	0.23	0.25	0.26	0.24	0.24

**Table 4:** Comparison of estimation accuracy in the five examined scenarios in Section 5.3.

	S11	S12	S13	S14	S15
mean	1.76	2.11	1.76	1.77	1.79
std	0.24	0.23	0.24	0.23	0.24

**Table 5:** Comparison of estimation accuracy in the last six examined scenarios in Section 5.4.

	S16	S17	S18	S19	S20	S21
mean	1.63	1.64	3.76	1.67	1.54	1.56
std	0.21	0.21	0.44	0.23	0.20	0.24

## Data Availability

Available upon request.

## Abbreviations

The following abbreviations are used in this manuscript:

LASSO	Least absolute shrinkage and selection operator
RGB	Red, green, and blue
ITU	International Telecommunication Union
DFT	Discrete Fourier transform
RMSE	Root mean square error
GP	Gaussian process
GT	Ground truth
